# First record of *Culex pipiens* (Diptera: Culicidae) in Alberta: expanding distributions and ecotype patterns in a western Canadian province

**DOI:** 10.1093/jme/tjae150

**Published:** 2024-12-20

**Authors:** Tiffany Pan, Michaela Seal, Hailey Shaw, Shahaanaa Mohanaraj, Gen Morinaga, Brittany Hogaboam, Michael Jenkins, Alexandra Coker, John Soghigian

**Affiliations:** Faculty of Veterinary Medicine, University of Calgary, Calgary, Alberta, Canada; Faculty of Veterinary Medicine, University of Calgary, Calgary, Alberta, Canada; Faculty of Veterinary Medicine, University of Calgary, Calgary, Alberta, Canada; Faculty of Veterinary Medicine, University of Calgary, Calgary, Alberta, Canada; Faculty of Veterinary Medicine, University of Calgary, Calgary, Alberta, Canada; Integrated Pest Management Lab, City of Edmonton, Edmonton, Alberta, Canada; Integrated Pest Management Lab, City of Edmonton, Edmonton, Alberta, Canada; Parks and Open Spaces, City of Calgary, Calgary, Alberta, Canada; Faculty of Veterinary Medicine, University of Calgary, Calgary, Alberta, Canada

**Keywords:** *Culex pipiens*, invasive species, ecotypes, distributions

## Abstract

*Culex pipiens* is an invasive mosquito found in temperate regions globally. It is considered among the most important disease vectors worldwide and is responsible for the transmission of a range of pathogens, including West Nile virus, avian malaria, Saint Louis encephalitis, and filarial worms. Throughout its northern temperate range, this mosquito is found in 2 ecotypes: form *pipiens* and form *molestus*. In Canada, this mosquito was previously thought restricted to the Pacific coast of British Columbia and the eastern provinces of Ontario, Quebec, and the Maritimes. Through routine mosquito surveillance and targeted trapping for *Cx. pipiens*, we detected this mosquito in 2 Albertan municipalities earlier than suggested by species distribution modeling based on climate change data. We confirmed the identity of putative *Cx. pipiens* specimens using DNA sequencing and found that alleles associated with form *molestus* were present, but at a low frequency compared to alleles associated with form *pipiens*. Furthermore, we compared the frequency of ecotype-related alleles in Alberta to elsewhere in North America and found a general trend of increased form *pipiens* in more northern latitudes, similar to previously reported results. We discuss our findings in the context of vector-borne disease activity in Canada, particularly West Nile virus.

## Introduction

Anthropogenic activities and the rise in global temperatures are contributing to the rapid expansion of insect vectors of disease (Fecchio et al. 2021). One such species is *Culex pipiens* Linnaeus 1758 (Diptera: Culicidae), the northern house mosquito or common house mosquito. This species is native to Europe, western Asia, and North Africa, but invasive in other temperate regions globally (Haba and McBride 2022). Its wide invasive distribution and the ability of this mosquito to be a successful vector of many pathogens such as West Nile virus, avian plasmodium, and nematodes (Turell et al. 2001) makes this species one of the most important disease vectors to humans and other animals in the northern hemisphere. It is widely considered responsible for the emergence of West Nile virus in North America in 1999 (Fecchio et al. 2021).

*Culex pipiens* is 1 member of a species complex, which includes *Cx. p. f. pipiens, Cx. p. f. molestus* Forskål 1775, *Cx. quinquefasciatus* Say 1823, *and Cx. australicus* Dobrotworsky and Drummond 1953 (Harbach 2012). These species are similar morphologically, which can make differential identification from morphology alone difficult, particularly when specimens are damaged by trapping. *Culex pipiens* is further divided into 2 ecotypes, form *pipiens* and form *molestus*, which we refer to collectively as *Cx. pipiens sensu stricto*, following Haba and McBride (2022). The females of these 2 ecotypes are similar in morphology but vary in ecology and can be differentiated via molecular markers (Becker et al. 2020). Form *pipiens* is primarily active above ground and feeds on birds (Haba and McBride 2022), while in northern latitudes, form *molestus* typically inhabits underground areas (e.g., sewers and train tunnels) and feeds on mammals, including humans (Haba and McBride 2022). Ecotypes appear to be fully reproductively compatible and can hybridize with other closely related species such as *Cx. quinquefasciatus* Say 1823 (Haba and McBride 2022). It is very likely that hybrids between *Cx. pipiens* ecotypes are more efficient vectors of disease and hybrids have even been associated with West Nile transmission in North America and Europe (Andreadis 2012, Turell 2012).

*Culex pipiens s.s.* has historically been restricted in Canada to southern Ontario, the Maritimes, and coastal British Columbia. However, species distribution models informed by climate change predictions indicated that by 2100, all southern Canada is expected to be an ideal habitat for *Cx. pipiens s.s.* (Hongoh et al. 2012). Moreover, recent habitat distribution models suggest suitable habitat exists in other provinces for this species (Gorris et al. 2021). *Culex pipiens* utilizes urban environments well, which will likely enable rapid expansion of this species as human development and climate change continue to alter the environment in Canada (Peach and Matthews 2022). Beyond its ability to utilize urban habitats, this species frequently oviposits in smaller temporary water sources, such as flowerpots, bird baths, and various other manmade containers (Haba and McBride 2022). This behavior exposes it to opportunities for human-aided dispersal, thus further increasing its potential for range expansion. Here, we document the first confirmations of *Cx. pipiens s.s.* in the province of Alberta through an initial detection of the species in Edmonton in 2018, and a subsequent detection in Calgary in 2022 through routine and targeted surveillance. We demonstrate via molecular methods that the populations found in this province are a mix of ecotypes and contextualize this within geospatial variation in ecotype frequencies across latitudes in North America.

## Materials and Methods

Due to the unexpected detection of *Cx. pipiens*, our surveillance efforts were reactive and not targeted towards this species. The first detection of *Cx. pipiens* specimen was during routine surveillance by the City of Edmonton in 2018. *Culex* mosquitoes that were abundant and looked markedly different from the native *Cx. tarsalis* Coquillett 1896 were set aside by mosquito surveillance technicians. These mosquitoes were identified morphologically as either *Cx. pipiens* or *Cx. restuans* Theobald, 1901. The finding of *Cx. pipiens* in Edmonton prompted collaboration with the City of Calgary to inspect specimens collected through their routine mosquito surveillance program for *Cx. pipiens*. In 2022, 4 CO_2_ traps and 6 New Jersey Light Traps were set up weekly from June to September in 6 different locations across Calgary, including city parks, depots, and landfills. Water buckets baited with hay-infused water were also set up in 4 locations from August to October and monitored 3 times a week for egg rafts, from which 3 egg rafts were collected and raised to larvae.

We identified both adult and larval *Cx. pipiens* from traps in Calgary in the summer of 2022 using morphological identification (Darsie and Ward 2005). We confirmed the morphological identifications of *Cx. pipiens* in Edmonton and Calgary with routine barcoding of an approximately 710 basepair fragment of the cytochrome oxidase subunit 1 gene (COX1) (Folmer et al. 1994), which we sequenced at the University of Calgary’s Centre for Health Genomics. We assembled the resulting sequencing reads in Geneious Prime (Geneious Prime 2023.0.4) and compared them to the NCBI nucleotide database with BLAST (Altschul et al. 1990). In addition, we used molecular “ecotyping” methods as described in Fonseca and Bahnck (2006) to characterize the ecotype of *Cx. pipiens* found in Alberta. In brief, we used previously published primers (Fonseca and Bahnck 2006) that targeted a region of the CQ11 microsatellite using a general forward primer and form-specific reverse primers that yield either a ~180 bp, for form pipiens, or a ~250 bp fragment for form *molestus*. The resulting fragments were visualized with a 2% agarose gel with a 100-bp size standard (Biotium).

To compare the frequency of *Cx. pipiens* ecotypes we found in Alberta to elsewhere in North America, we conducted a literature review of *Cx. pipiens* ecotypes across North America. We searched Google Scholar with the keywords “North America” and “CQ11,” “Canada” and “CQ11,” and “United States” and “CQ11”. We only selected studies that included *Cx. pipiens* specimens collected in North America and molecularly analyzed using CQ11 primers. Nine papers met our criteria and provided enough information for analysis (Supplementary Table 1). We extracted the sampling location, microhabitat (i.e., above or below ground), and the proportion of form *pipiens* and form *molestus* specimens from each article. We also included data from the molecular analysis of Alberta specimens from Calgary and Edmonton in the analysis. We calculated the frequency of each ecotype for each location, and geospatial analyses of the data were completed in R version 4.3.2 using *ggplot2* (v3.4.4; Wickham 2024).

## Results and Discussion

We confirmed the identity of *Culex* mosquitoes collected in Edmonton in 2018 as *Cx. pipiens* (Supplementary Table 2). In Calgary, we first detected this mosquito in 2022 (Supplementary Table 2). Due to the large volume of mosquitoes collected (>93,000) in 2022 in Calgary, we identified a subset of 15% of the collection. A total of 15 *Cx. pipiens* were found in 2 landfill locations in Calgary in 4 traps during August. Across the season, 13 *Aedes* species, 2 *Culex* species, and 4 *Culiseta* species were collected. Of the specimens identified, the majority were *Aedes*. Future work will investigate the efficiency of different trap types for collecting *Cx. pipiens*. Since its first detection in both municipalities, the mosquito has become widespread in both municipalities and is commonly found during surveillance activities. Unfortunately, as our sampling was primarily reactive, we are unable to judge exactly how fast the mosquito has expanded throughout both cities.

A BLAST analysis of the fragment of COX1 we sequenced showed that Alberta *Cx. pipiens* specimens had a high pairwise identity, ranging from 99.69% to 100%, to North American *Cx. pipiens* sequences (Supplementary Table 2). However, this may not indicate the geographic origin of our invasive population, as the pairwise identity for this region of COX1 is very high for *Cx. pipiens* across the invasive and putative native range of this species. In total, we sequenced 30 *Cx. pipiens* mosquitoes via barcoding (Table 1) and we deposited representative sequences from both municipalities in GenBank under ascensions PQ601625- PQ601630 (Supplementary Table 2).

**Table 1.** Molecular analysis of alleles associated with ecotype and total samples sequenced via COX1

City	Collection year	Sample total	% with 2 *pipiens* alleles	% with 2 *molestus* alleles	% 1 *pipiens* and 1 *molestus* allele)	# Tested with COX1
Calgary	2022	30	80	6.7	13.3	25
Edmonton	2018, 2020, 2021	173	80.3	3.5	16.2	5

**Fig. 1.**
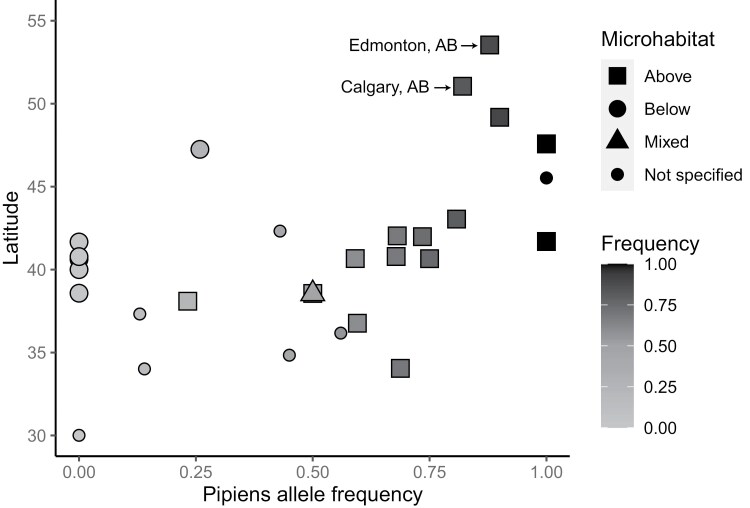
Frequency of *pipiens* alleles across latitude and microhabitat for *Culex pipiens* found in North America based on analysis with CQ11 primers from 2006 to 2022. *Culex pipiens* sampled from Calgary and Edmonton, Alberta, Canada, most commonly had the *pipiens* allele, but with a notable percentage of *molestus* alleles (see Table 1).

We confirmed that alleles associated with both ecotypes, form *pipiens* and form *molestus*, are present in Edmonton and Calgary (Table 1) based on analysis with the CQ11 primers. In total, we tested 203 *Cx. pipiens* mosquitoes using the CQ11 primers, which included the mosquitoes we previously barcoded (Table 1). Alberta populations were dominated by the form *pipiens* allele, which is consistent with other above-ground populations at more northerly latitudes (Figure 1 and Supplementary Table 1). This reflects the general trend of increasing form *pipiens* allele frequency in above-ground populations as latitude increases, a phenomenon also observed in European populations (Haba and McBride 2022), although we caution direct interpretation of *pipiens* and/or *molestus* frequencies in unsampled populations at particular low latitudes due to the presence of *Cx. quinquefasciatus* in North America.

Our finding of a small percentage of *Cx. pipiens* with form *molestus*-associated alleles in Alberta is surprising, as neither city has significant underground subway systems, which are commonly associated with this form. However, *molestus* alleles at the CQ11 locus may not indicate mosquitoes of form *molestus* are in fact found in Alberta; it is possible that the signature of *molestus* in the mosquitoes in Alberta was a result of admixture at some point in the past prior to arrival in the province. Alternatively, it is possible that form *molestus* mosquitoes are found in Alberta, as other underground habitats exist in the province, including light rail systems that travel briefly underground in both cities, as well as substantial numbers of underground parking garages, which may serve as habitat for these mosquitoes. Whether historical admixture or present populations of *molestus* mosquitoes are responsible for the small signature of *molestus* we have detected in Alberta requires further study, such as through surveillance in underground habitats and genomic analyses to give a more comprehensive picture of the genetic background of Alberta *Cx. pipiens*.

As global temperatures rise, vector-borne diseases pose an increased risk as mosquitos such as *Cx. pipiens* expand their range (Fecchio et al. 2021). Additionally, increasing global temperatures will promote the development of mosquito larvae (Becker et al. 2020) and may increase feeding rates and reproduction of adult vectors (Mordecai et al. 2017). Owed to its vector competence for West Nile virus, *Cx. pipiens* is of particular concern as climate change is expected to triple West Nile virus cases in the continental United States over the next 30 years (Paull et al. 2017). West Nile virus was first detected in Canada in 2001 with particularly high cases in 2003 and 2007 (Giordano et al. 2017, Public Health Agency of Canada 2024). West Nile virus now causes only sporadic human and horse cases in Canada, and as a result, surveillance for both the vectors and the pathogen has declined throughout much of Canada. In light of previously reported expansion of invasive mosquito species in Canada (Peach and Matthews 2022), and the expansion we report here for *Cx. pipiens*, a review and potential expansion of vector and virus surveillance may be warranted in Canada generally. Furthermore, *Cx. pipiens* is an efficient vector for avian malaria. Although typically thought to be largely harmless to native birds in North America, a new virulent strain of avian malaria has recently been detected in the United States (Theodosopoulos et al. 2021). In addition, exotic birds are at risk—for instance, there have already been deaths recorded of 2 zoo penguins in Calgary (Wilder Institute/Calgary Zoo 2023), leading to increased concern of the risks *Cx. pipiens* poses to exotic and native birds in Alberta.

As *Cx. pipiens* is considered a major global disease vector, its expansion into Alberta warrants further study. Whether this mosquito poses a significant risk to veterinary and public health in the province is presently unknown due to our lack of knowledge regarding its arrival and ecology in the province. To address this, future studies characterizing the ecology of *Cx. pipiens* in an Albertan context should be undertaken, as well as genetic studies to assess the origin of populations found here. Such studies will provide important insight into the risk this mosquito brings to Alberta and elsewhere.

## Supplementary data

Supplementary data are available at *Journal of Medical Entomology* online.

tjae150_suppl_Supplementary_Material
