# Supplementary material for: First record of Culex pipiens (Diptera: Culicidae) in Alberta: expanding distributions and ecotype patterns in a western Canadian province
Source: J Med Entomol. 2024 Dec 20;62(2):461–4. doi: 10.1093/jme/tjae150 (PMC11919614; doi:10.1093/jme/tjae150)
Supplement: tjae150_suppl_Supplementary_Material [file tjae150_suppl_supplementary_material.pdf]

Supplementary Material for Pan et al., "First record of *Culex pipiens* (Diptera: Culicidae) in Alberta: Expanding distributions and ecotype patterns in a western Canadian province"

Supplementary Table 1. List of sources used for the analysis of ecotype frequencies across North America and the sampling location used in each source. Only sources that used CQ11 primers for ecotype analysis were used, and papers found were published between 2006 and 2016.

**Source**	**Sampling location**
Kent, R.J., Harrinton, L.C. & Norris, D.E. (2007)	New York
Cornel, A., Lee, Y., Fryxell, R.T., Siefert, S., Nieman, C., Lanzaro, G. (2012)	California
Bahnck, C.M. & Fonseca, D.M. (2006)	Massachusetts, California, South Carolina, Louisiana, Pennsylvania, Oregon, North Carolina
Chaulk, A.C., Carson, K.P., Whitney, H.G., Fonseca, D.M., & Chapman, T.W. (2016)	Newfoundland
Asgharian, H., Chang, P.L., Lysenkov, S., Scobeyeva, V.A., Reisen, W.K., & Nuzhdin, S.V. (2015)	Sacramento, CA
Fritz, M.L., Walker, E.D., Miller, J.R., Severson, D.W., & Dworkin, I. (2015)	Illinois
Kothera, L., Godsey, M., Mutebi, J., & Savage, H. (2010)	New York

Supplementary Table 2. A subsample of sequenced mosquito specimens with their first top North American BLAST results. Specimens were collected from Edmonton and Calgary from 2018 and 2022 respectively.

Lab Code	Accession	City	Year Collected	Sequence Length	Top BLAST Result	Percent Identity	Accession Match
047ED	PQ601628	Edmonton	2018	643	*Culex pipiens*	100%	PP558242.1
049ED	PQ601629	Edmonton	2018	643	*Culex pipiens*	99.69%	PP558242.1
050ED	PQ601630	Edmonton	2018	638	*Culex pipiens*	99.84%	PP558242.1

010CA	PQ601625	Calgary	2022	643	*Culex pipiens*	100%	PP558242.1
011CA	PQ601626	Calgary	2022	643	*Culex pipiens*	99.85%	PP558242.1
020CA	PQ601627	Calgary	2022	645	*Culex pipiens*	100%	PP558242.1

Supplementary Table 3. List of locations used for the spatial analysis of North America ecotypes and their associated microhabitat (above or below ground, where specified) and the percentage of *pipiens* and *molestus* alleles found in each sample.

**Collection location**	**Microhabitat**	***Pipiens* allele frequency (%)**	***Molestus* allele frequency (%)**
New York, NY	Above	59.1	40.9
New York, NY	Below	0.0	100.0
New York, NY	Above	67.9	32.1
New York, NY	Above	75.0	25.0
Syracuse, NY	Above	80.8	19.2
91st St sewer, New York, NY	Below	0.0	100.0
Marin, CA	Above	23.3	76.7
Sacramento, CA	Above	50	50
Sacramento, CA	Below	0.0	100.0
Sacramento, CA	Mixed	50.0	50.0
Fresno, CA	Above	59.6	40.4
Los Angeles, CA	Above	68.8	31.2
Boston, MA	Not specified	43.0	57.0
Chino, CA	Not specified	14.0	86.0
Greenville, SC	Not specified	45.0	55.0
New Orleans, LA	Not specified	0.0	100.0
Philadelphia, PA	Below	0.0	100.0
Portland, OR	Not specified	100.0	0.0
San Jose, CA	Not specified	13.0	87.0
Walkertown, NC	Not specified	56.0	44.0
St John's, NL	Above	100.0	0.0
Deer Lake, NL	Above	90.0	10.0
Calumet, IL	Below	25.9	74.1
Oak Lawn, IL	Above	100.0	0.0
Chicago, IL	Above	68.1	31.9
Chicago, IL	Above	73.5	26.5
Chicago, IL	Below	0.0	100.0
Calgary, AB	Above	82.1	17.9
Edmonton, AB	Above	87.9	12.1

Supplementary References

Asgharian H, Chang PL, Lysenkov S, Scobeyeva VA, et al. 2015. Evolutionary genomics of Culex pipiens: global and local adaptations associated with climate, life-history traits and anthropogenic factors. Proc. R. Soc., Ser. B, Biol. Sc. 282(1810):20150728. https://doi.org/10.1098/rspb.2015.0728.

Bahnck CM, Fonseca DM. 2006. Rapid assay to identify the two genetic forms of Culex (Culex) pipiens L.(Diptera: Culicidae) and hybrid populations. Am. J. Tropical Med. Hyg. 75(2):251-5.

Chaulk AC, Carson KP, Whitney HG, et al. 2016. The arrival of the northern house mosquito Culex pipiens (Diptera: Culicidae) on Newfoundland's Avalon Peninsula. J. Med. Entomol. 53(6):1364-9. https://doi.org/10.1093/jme/tjw105.

Cornel A, Lee Y, Fryxell RT, et al. 2012. Culex pipiens sensu lato in California: a complex within a complex?. J. Am. Mosq. Control Association. 113-21. https://doi.org/10.2987/8756-971X-28.4s.113.

Fritz ML, Walker ED, Miller JR, et al. 2015. Divergent host preferences of above-and below-ground C ulex pipiens mosquitoes and their hybrid offspring. Med. Veterinary Entomol. 29(2):115-23. https://doi.org/10.1111/mve.12096.

Kent RJ, Harrington LC, Norris DE. 2007. Genetic differences between *Culex pipiens f. molestus* and *Culex pipiens pipiens* (Diptera: Culicidae) in New York. J. Med. Entomol. 44(1):50-9. https://doi.org/10.1093/jmedent/41.5.50.

Kothera L, Godsey M, Mutebi JP, Savage HM. 2010. A Comparison of Aboveground and Belowground Populations of *Culex pipiens* (Diptera: Culicidae) Mosquitoes in Chicago, Illinois, and New York City, New York, Using Microsatellites. J. Med. Entomol. 47(5):805-13. https://doi.org/10.1093/jmedent/47.5.805.
